# Cardiometabolic thresholds for peak 30-min cadence and steps/day

**DOI:** 10.1371/journal.pone.0219933

**Published:** 2019-08-02

**Authors:** Bryan Adams, Katie Fidler, Noah Demoes, Elroy J. Aguiar, Scott W. Ducharme, Aston K. McCullough, Christopher C. Moore, Catrine Tudor-Locke, Diana Thomas

**Affiliations:** 1 Department of Mathematical Sciences, United States Military Academy, West Point, New York, United States of America; 2 Department of Kinesiology, School of Public Health and Health Sciences, University of Massachusetts Amherst, Amherst, Massachusetts, United States of America; University of Maiduguri College of Medical Sciences, NIGERIA

## Abstract

**Purpose:**

To provide empirically-supported thresholds for step-based intensity (i.e., peak 30-min cadence; average of the top 30 steps/min in a day) and steps/day in relation to cardiometabolic health outcomes.

**Methods:**

Receiver operating characteristic curve analysis was applied to the National Health and Nutrition Examination Survey (NHANES) 2005–2006 accelerometer-derived step data to determine steps/day and peak 30-min cadence as risk screening values (i.e., thresholds) for fasting glucose, body mass index, waist circumference, high blood pressure, triglycerides, and HDL cholesterol. Thresholds for peak 30-min cadence and steps/day were derived that, when exceeded, classify the absence of each cardiometabolic risk factor. Additionally, logistic regression models that included the influence of age and smoking were developed using the sample weights, primary sampling units (PSUs), and stratification variables provided by the NHANES survey. Finally, a decision tree analysis was performed to delineate criteria for at-risk versus healthy populations using cadence bands.

**Results:**

Peak 30-min cadence thresholds across cardiometabolic outcomes ranged from 66–72 steps/min. Steps/day thresholds ranged from 4325–6192 steps/day. Higher thresholds were observed in men compared to women. In men, higher steps/day thresholds were observed in age ranges of 30–39, while in women, higher thresholds were observed in the age-range 50–59 years. Decision trees for classifying being at low risk for metabolic syndrome contained one risk-free leaf at higher cadence bands, specifically for any time accumulated at ≥120 steps/min.

**Conclusions:**

Minimum thresholds representing absence of cardiometabolic risk range from 4325–6192 steps/day and 66–72 steps/min for peak 30-min cadence. Any time accumulated at ≥120 steps/min was associated with an absence of cardiometabolic risk. Although based on cross-sectional data, these thresholds represent potentially important and clinically interpretable daily physical activity goals.

## Introduction

The number of cases of metabolic syndrome in U.S. adults are on the rise, with a current estimated prevalence of 35% [1]. Since the presence of metabolic syndrome is known to increase the risk of cardiovascular events and all-cause mortality [2, 3], there is a growing need for the prevention, diagnosis, and treatment of this chronic disease.

Physical inactivity is an established modifiable risk factor for metabolic syndrome [4]. In response, national physical activity guidelines have been developed communicating the dose (e.g. volume, frequency, and intensity) associated with preventing and treating metabolic syndrome [5]. The 2018 Physical Activity Guidelines Advisory report [6] specifically targeted daily step counts (steps/day) as a publicly consumable metric for measuring and prescribing physical activity volume. Additionally, a strong relationship between cadence (steps/min) and absolutely-defined physical activity intensity (e.g., metabolic equivalents; METs), has been demonstrated in laboratory-based studies (*r* = 0.93) [7]. The application of cadence combined with daily step counts is particularly attractive since it is easily tractable from the same time-stamped accelerometry devices used to report daily step counts [8]. Cadence patterns can be further distilled by averaging the cadence values of the 30 highest (but not necessarily consecutive) minutes in a day, and averaging these values over one week, providing an index metric known as “peak 30-min cadence” [9]. We note that the NHANES step data are reported as step counts accumulated in 1-min epochs (i.e., steps per minute) and represents an average cadence over a one-minute interval and thus is only an approximation of instantaneous cadence [10].

The peak 30-min cadence metric represents not only the best effort intensity for a given day, but also the persistence of highest-intensity behavior across a week. Together, steps/day and peak 30-min cadence can provide simple and understandable translations of physical activity volume and intensity measurements, which may then be associated with risk of developing chronic diseases, including metabolic syndrome.

Reliance on step data to deliver prescriptions requires rigorous data driven analysis that provide steps/day and peak 30-min cadence thresholds associated with specific health outcomes [6]. Some dose-response relationships have been identified between steps/day and metabolic syndrome [11], but to date analysis strategies have solely focused on daily steps counts.

Here, using the National Health and Nutrition Examination Survey (NHANES) 2005–2006 dataset, we for the first time use a receiver operating characteristic and decision tree analysis to derive clinically interpretable thresholds for steps/day and peak 30-min cadence for numerous cardiometabolic risk factors. The derived thresholds provide minimum bounds on steps/day and peak 30-min cadence associate with the absence of each risk factor. We also identify time spent in sedentary to higher intensity activities [8] that are associated with complete absence of any risk factor which can also be thought of as a threshold goal associated with ideal health status.

## Methods

### Data source

The participant data included in this analysis were sourced from the NHANES 2005–2006 Physical Activity Monitor (PAM) dataset. The purpose of NHANES is to assess the health, nutritional and physical activity status of noninstitutionalized adults and children in nationally representative sample from the United States. A detailed description of the NHANES PAM protocols are available online (http://www.cdc.gov/nchs/data/nhanes/nhanes_05_06/BM.pdf), and a catalog of variable definitions and data treatment rules has been assembled and reported elsewhere [12]. All NHANES protocols were approved by The National Center for Health Statistics ethics review board. Participants were required to provide informed consent. In the 2005–2006 survey cycle, physical activity was objectively measured using the hip worn ActiGraph 7164 accelerometer (ActiGraph, Ft Walton Beach, Florida). In addition to the more conventional activity count output, the ActiGraph 7164 also provides an enumeration of steps taken (stored in 1-min epochs), allowing for the calculation of steps/day, peak 30-min cadence and time spent in different cadence bands (defined below).

### Data treatment

#### Accelerometry

The NHANES 2005–2006 database was prepared for analysis using the software package R (**R** Core Team (2013)). We first restricted the database to adults 18 years or older. Non-wear time was defined as 60 consecutive minutes of zero accelerometer counts/min, with a valid day defined as ≥ 10 hours [12]. Any participant without 4 valid wear days [12] was removed from the dataset. The NHANES protocol requested participants wear the accelerometer for a 7-day period and in this way ensure that a weekend is included. Restricting to a minimum of 4 valid wear days may lose weekend wear time in some of the subjects. Using the standard definition of non-wear time (i.e. 60 consecutive minutes of zero counts/min [12]), The R package “dplyr” was used to remove all non-wear time from the database. The consecutive 60 minutes of non-wear time was determined by the accelerometer count equaling zero with a rolling sum of the difference in accelerometer counts between minutes for each subject.

Data were removed when the difference did not change for 60 minutes and the step count equaled zero. Next, only recorded minutes that were deemed reliable by the NHANES team under the NHANES identifier PAXSTAT and when the physical activity monitor (PAM) was calibrated (PAXCAL) were retained [13]. We also removed (i.e., censored) steps associated with intensity levels less than 500 activity counts/min as per recommended convention to make the data more scalable to pedometer-based output [14]. Without removing the steps associated with intensity levels less than 500 activity counts/min, the total steps/day are high and implausible as indicated in [14]. Finally, any data over 180 steps/min were also removed. These data were removed after comparison of the PAM’s output of intensity with the output of cadence (S1 Fig—a scatter plot comparing steps/min with the PAM’s activity-count based intensity output). Of note is that, when cadence exceeds 180 steps/min, the step count increases as the activity-count based intensity decreases, which is implausible. This observation was also supported by Rowlands et al. [15], who demonstrated that the ActiGraph GT1M underestimated the step count at higher speeds.

#### Cardiometabolic risk factor thresholds

We applied published thresholds to classify risk cut-offs for BMI, waist circumference, high-density lipoprotein (HDL) cholesterol, triglycerides, systolic blood pressure (SBP), diastolic blood pressure (DBP), high blood pressure, and fasting glucose (Table 1). In some cases (e.g., waist circumference or blood pressure) there are low-risk and high-risk thresholds. For these variables, the low-risk and high-risk thresholds were independently analyzed, thereby generating a low-risk and a high-risk thresholds for peak 30-min cadence and steps/day. Additionally, each participant was assessed for metabolic syndrome, defined as a diagnosis of exceeding at least three of the five metabolic risk thresholds [16] (Table 1).

**Table 1:** Thresholds for different metabolic related health parameters.

Risk Factor	Threshold values/s	
BMI [33]	Low-risk (overweight): BMI ≥25.00 kg/m^2^	High-risk (Class I obesity) BMI ≥30.00 kg/m^2^
Waist circumference [33]	Males Low-risk ≥ 94 cm Females Low-risk ≥80 cm	Males High-risk ≥102 cm Females High-risk ≥88 cm
Systolic blood pressure [34]	Low-risk ≥120 mmHg	High-risk ≥130 mmHg
Diastolic blood pressure [34]	Low-risk ≥80 mmHg	High-risk ≥85 mmHg
High Blood Pressure [34]	Systolic Blood Pressure 130 mm Hg and/or Diastolic Blood Pressure 85 mm Hg	
Fasting Glucose [35]	≥100 mg/dL	≥126 mg/dL
HDL cholesterol [36]	Low-risk ≤ 59 mg/dL	High-risk ≤ 40 mg/dL
Triglyceride [36]	Low-risk ≥150 mg/dL High-risk ≥200 mg/dL	
Metabolic Syndrome 3 of the 5 [16]	High-risk waist circumference	
	Triglycerides 150 mg/dL	
	HDL cholesterol: Males 40 mg/dL, Females 50 mg/dL	
	High Blood Pressure: Systolic Blood Pressure 130 mm Hg and/or Diastolic Blood Pressure 85 mm Hg	
	Fasting glucose 100 mg/dL	

Subject characteristics after data processing appear in Table 2.

**Table 2:** Subject characteristics of NHANES data after processing.

	Male Mean (SD)	Female Mean (SD)
**Age (yr)**	49.02 (19.65)	47.35 (19.51)
**Peak 30 steps/min**	70.14 (21.57)	66.24 (23.91)
**steps/day**	6933.1 (4042.25)	5390.23 (3117.79)
**Waist Circumference (cm)**	99.63 (15.13)	95.18 (15.54)
**Triglycerides (mg/dL)**	152.1 (122.37)	132.12 (88.3)
**HDL cholesterol (mg/dL)**	49.18 (13.63)	60.28 (15.96)
**Diastolic BP (mmHg)**	70.47 (12.22)	67.7 (12.34)
**Systolic BP (mmHg)**	124.3 (16.36)	121.82 (20.4)
**Fasting glucose (mg/dL)**	107.11 (31.62)	103.43 (35.24)
**BMI (kg/m^2)**	28.08 (6.09)	28.88 (7.08)

### Statistical analysis

#### Design

Three main questions drove this study analysis.

1. What are the minimum steps/day and peak 30-min cadence (steps/min) that one should achieve to avoid exhibition of each specific cardiometabolic risk factor? These thresholds theoretically represent a minimum target value below which an individual should attempt to not fall.
2. How does age or gender affect the thresholds in Question 1? Specifically, do the thresholds remain uniform or are they age or gender dependent?
3. At what cadence and how much time spent at this cadence can individuals be classified as healthy, that is, enjoying an absence of all cardiometabolic risk factors tested. These thresholds theoretically represent an upper target value for more optimal health.

To address these three questions, we used the publicly available NHANES 2005–2006 database that contains minute-by-minute step accumulation data along with measured waist circumference, high-density lipoprotein (HDL) cholesterol, triglycerides, systolic blood pressure (SBP), diastolic blood pressure (DBP), high blood pressure, and fasting glucose.

In reference to Question 1, we employed a receiver operating characteristic (ROC) analysis to determine the capacity of peak 30-min cadence and steps/day to classify individuals in the NHANES dataset for the different cardiometabolic risk factors.

The second question was evaluated by grouping participants into 10-year age ranges and performing the ROC analysis to determine age-specific classification thresholds. This analysis was performed to facilitate clinical interpretation and application of the thresholds. We also developed logistic regression models with steps/day and peak 30-min cadence, both adjusted by age and smoking. Each logistic regression model used the sample weights, PSUs, and stratification variables provided by the NHANES survey team [17]. While the quality of the model can be assessed, the thresholds are not clinically interpretable and so we report instead the odds ratio (OR).

Finally, Question 3 was evaluated using a decision tree analysis that classified participants into healthy versus at risk groups based on time spent in cadence bands [8]. This analysis provides information regarding the required time spent in derived cadence bands associated with the absence of risk.

#### ApproachReceiver operating characteristic curve analysis

A ROC curve analysis was conducted using the statistical program R (**R** Core Team (2013)). The R package ‘dplyr’ was used to group and filter the NHANES data by age and to assign the binary outputs of 0 if the designated metabolic health risk threshold (Table 1) was not met and 1 if the standard was met. The R package, pROC was then used to classify true positives, false positives, true negatives, and false negatives for each disease state described in Table 1. The pROC package outputs the threshold, which simultaneously maximizes true positives and minimizes false negatives and the resulting area under the curve (AUC) of the ROC curve. Ninety-five percent confidence intervals for the AUC and thresholds were also provided by the pROC package.

### Logistic regression model

The R package ‘survey’ was used to perform logistic regression. The survey package takes into account the sample weights, PSUs, and stratification variables provided by the NHANES survey [17]. We tested the necessary assumption that the logit was linear in each continuous covariate (peak 30-min cadence and age).

Since peak 30-min cadence is a continuous covariate, reporting the Odds Ratio (OR) for the increase of 1 step/min in a peak 30-min cadence would be of little interest. Instead, each OR was calculated using an increase of 20 steps/min to match the 20 steps/min discretization of cadence bands, $OR=e^{20\hat{\beta }}$, subsequently the 95% confidence intervals are reported using ${(e}^{20\hat{\beta }\pm 1.96\times 20\times \hat{SE}(\hat{\beta )}})$. The choice of 20 steps/min was selected because this is equivalent to moving the peak 30-min cadence up one cadence band [18]. Accordingly, ORs for peak 30-min cadence can be interpreted as “for every increase in 20 steps/min, an individual is [OR] times less likely to be at risk for the respective negative metabolic health outcome.” Similar to the ORs for peak 30-min cadence, steps/day ORs were calculated using a scalar of 1000 steps/day.

#### Thresholds by age ranges

The data were grouped by age ranges [18,29], [30, 39], [40,49], [50,59], [60,69] and [70,85]. Sex-specific ROC curve analyses were performed using the data in each age strata. The resulting AUC and l thresholds were calculated for each sex and age strata.

#### Decision tree analysis of cadence bands

A decision tree analysis was performed using the Classification and Regression Tree (CART) algorithm through the R package, “rpart” [19]. The CART algorithm uses input variables to classify an outcome; in our case a binary outcome of either having or not having the cardiometabolic risk factor. The algorithm is sometimes referred to as recursive partitioning because the goal of the process is to partition the data into groups iteratively until no predictive improvement is achieved when further partitioning the group. More specifically, the original dataset is first split into two groups using the optimal variable, i.e., the variable that decreases the risk the most. One group is generated with the lowest risk and the other group represents the remainder of the dataset. The same process is then applied again separately to these two new groups, breaking them into further subgroups until either the algorithm has reached a minimum group size or if no more improvements can be made in predicting whether the group can be further delineated into at-risk versus risk-free categories [20].

The NHANES step data were first separated into cadence bands representing the total number of minutes spent at: 0 steps/min (zero cadence; non-movement during wear time), 1–19 steps/min (incidental movement), 20–39 steps/min (sporadic movement), 40–59 steps/min (purposeful stepping), 60–79 steps/min (slow walking), 80–99 (medium walking), 100–119 (brisk walking) and ≥120 steps/min (all faster ambulation) [8]. The amount of time spent in each cadence band was averaged over valid days of wear. The time spent in cadence bands were then used as model inputs to classify individuals as above or below the low risk metabolic syndrome threshold. The decision tree classification was repeated 1000 times by assigning 80% of the data as training dataset and 20% of the data reserved to evaluate the cross-validated classification accuracy. The decision tree analysis was performed in the statistical package R (**R** Core Team (2013)) using the “rpart” (Recursive Partitioning and Regression Trees) package.

## Results

### Participants

The entire dataset prior to data cleaning consisted of 10348 individuals, of whom 5080 were men and 5268 were women. From the full dataset, 3377 participants had valid accelerometer data. To identify the existence of metabolic syndrome concomitant metabolic measurements of waist circumference, triglycerides, HDL cholesterol, systolic and diastolic blood pressure and fasting glucose are required. Of the 3377 participants, there were a total of 1065 participants who had all five complete metabolic measurements available to determine whether or not they had metabolic syndrome. Out of the participants with valid steps/day and peak 30-min cadences, 178 were classified with “high-risk” for metabolic syndrome, 1742 were classified without “high-risk” metabolic syndrome, 1143 were classified with “low-risk” metabolic syndrome, and 706 were classified without “low-risk” metabolic syndrome. The remainder of the participants could not be classified as “high-risk” (1457 participants) or “low-risk” (1528 participants) because of missing measurements. Average BMI in the final analytical sample was 28.47 ± 6.60 kg/m^2^ with mean age 48.15 ± 19.51 years.

#### Thresholds for peak 30-min cadence and steps/day

All AUC values for both peak 30-min cadence and steps/day were greater than or equal to 0.50 (Tables 3 and 4). The highest AUC values were associated with high-risk fasting glucose (AUC = 0.701 for steps/day) and high-risk metabolic syndrome (AUC = 0.68 for peak 30-min cadence). The lowest AUCs were observed for high-risk HDL cholesterol (AUC = 0.50 for steps/day) and low-risk HDL cholesterol (AUC = 0.51 for peak 30-min cadence) with the lower bound of the confidence interval below 0.50. Peak 30-min cadence ranged from 66–72 steps/min across cardiometabolic risk factors. Thresholds for steps/day ranged from 4325–6192 steps/day.

**Table 3:** Peak 30-min cadence, AUC and thresholds to classify each of the known cardiometabolic risk factors. Peak 30-min cadence above the threshold classifies positive health outcomes.

Response	AUC	Peak 30-min cadence threshold (steps/min)	Sensitivity	Specificity	Controls	Cases
Metabolic Syndrome Low-risk	0.65	69.62	0.64	0.59	706	1143
	[0.62, 0.67]	[67.69, 71.40]				
Blood Pressure Low-risk	0.60	67.69	0.64	0.53	1133	1235
	[0.58, 0.63]	[66.23, 71.21]				
Waist Circumference Low-risk	0.65	71.87	0.63	0.60	858	2425
	[0.63, 0.67]	[70.63, 74.24]				
Triglycerides Low-risk	0.57	67.59	0.59	0.54	1035	500
	[0.54, 0.60]	[66.53, 71.41]				
HDL Low-risk	0.52	69.12	0.53	0.49	1079	2161
	[0.59, 0.54]	[69.11, 74.75]				
Fasting Glucose Low-risk	0.60	66.01	0.65	0.52	851	697
	[0.57, 0.63]	[63.55, 71.20]				
BMI Low-risk	0.57	72.06	0.52	0.58	1087	2273
	[0.54, 0.59]	[68.81, 74.33]				
Metabolic Syndrome High-risk	0.68	66.20	0.64	0.68	1742	178
	[0.64, 0.72]	[61.40, 67.65]				
Blood Pressure High-risk	0.64	66.81	0.64	0.58	1618	750
	[0.61, 0.66]	[64.97, 70.33]				
Waist Circumference High-risk	0.64	68.20	0.62	0.61	2065	1218
	[0.62, 0.66]	[66.00, 71.08]				
Triglycerides High-risk	0.55	67.47	0.56	0.54	1242	293
	[0.51, 0.59]	[64.15, 72.23]				
HDL High-risk	0.52	72.49	0.45	0.60	2641	599
	[0.50, 0.55]	[66.92, 74.25]				
Fasting Glucose High-risk	0.68	62.00	0.67	0.63	1396	152
	[0.63, 0.73]	[55.25, 65.88]				
BMI High-risk	0.61	69.11	0.58	0.60	2238	1122
	[0.59, 0.63]	[66.67, 71.11]				

**Table 4:** Steps/day, AUC, sensitivity, specificity and thresholds that delineate cardiometabolic risk factors. Steps/day below the threshold classifies the at-risk population. The sample size and number of at-risk cases for each ROC analysis are provided.

Response	AUC	Steps/day threshold	Sensitivity	Specificity	Controls	Cases
Metabolic Syndrome Low-risk	0.61	5508	0.63	0.53	706	1143
	[0.58, 0.63]	[5113, 5834]				
Blood Pressure Low-risk	0.58	5247	0.637	0.496	1133	1235
	[0.56, 0.60]	[4907, 5834]				
Waist Circumference Low-risk	0.64	6308	0.59	0.618	858	2425
	[0.621, 0.663]	[5696, 6845]				
Triglycerides Low-risk	0.56	5518	0.564	0.546	1035	500
	[0.53, 0.59]	[5076, 5618]				
HDL Low-risk	0.51	6192	0.58	0.467	1079	2161
	[0.49, 0.53]	[5992, 6432]				
Fasting Glucose Low-risk	0.58	5111	0.63	0.496	851	697
	[0.55, 0.60]	[4714, 5913]				
BMI Low-risk	0.54	5580	0.58	0.509	1087	2273
	[0.52, 0.56]	[5199, 5918]				
Metabolic Syndrome High-risk	0.69	4908	0.67	0.64	1742	178
	[0.65, 0.73]	[4222, 5501]				
Blood Pressure High-risk	0.62	5247	0.63	0.57	1618	750
	[0.60, 0.65]	[4740, 5746]				
Waist Circumference High-risk	0.66	5685	0.6	0.64	2065	1218
	[0.64, 0.68]	[5162, 5992]				
Triglycerides High-risk	0.54	5336	0.57	0.52	1242	293
	[0.51, 0.58]	[4908, 5564]				
HDL High-risk	0.50	5848	0.51	0.50	2641	599
	[0.47, 0.53]	[5451, 6786]				
Fasting Glucose High-risk	0.701	4325	0.69	0.63	1396	152
	[0.66, 0.75]	[3677, 5077]				
BMI High-risk	0.58	5628	0.56	0.58	2238	1122
	[0.56, 0.60]	[5112, 5766]				

#### Logistic regression models

Four different models were created to assess the impact of peak 30-min cadence and steps/day to estimate thresholds for waist circumference, blood pressure, and metabolic syndrome. One model included peak 30-min cadence or steps/day as univariate predictors, and the remaining models were adjusted for age and smoking. Each model’s Akaike information criterion (AIC) was compared to select the best model (see S5 Table for AIC’s for each model). In all cases, the model that adjusted for age and smoking had the lowest or nearly lowest AIC. As such, these are the reported OR’s.

Table 5 reports OR values for all cases where the results were statistically significant. OR ranged from 1.18 to 1.60 with the highest OR occurring in peak 30-min cadence adjusted for age and smoking classifying cardiometabolic risk. The 95% CI for the OR in adjusted peak 30-min cadence classifying high-risk metabolic syndrome was (1.13, 2.04).

**Table 5:** Odd ratios derived from the logistic regression model with peak 30-min cadence and steps/day adjusted for age and smoking.

	Waist Circumference (cm)	Blood Pressure (mm Hg)	Metabolic Syndrome
Peak 30-min cadence high-risk	1.60 [1.31,1.96]	1.27 [1.19,1.63]	1.53 [1.13,2.04]
Peak 30-min cadence low-risk	1.43[1.23,1.68]	1.18 [1.03,1.36]	1.54 [1.32,1.79]

#### Thresholds by age ranges

**Fig 1:**
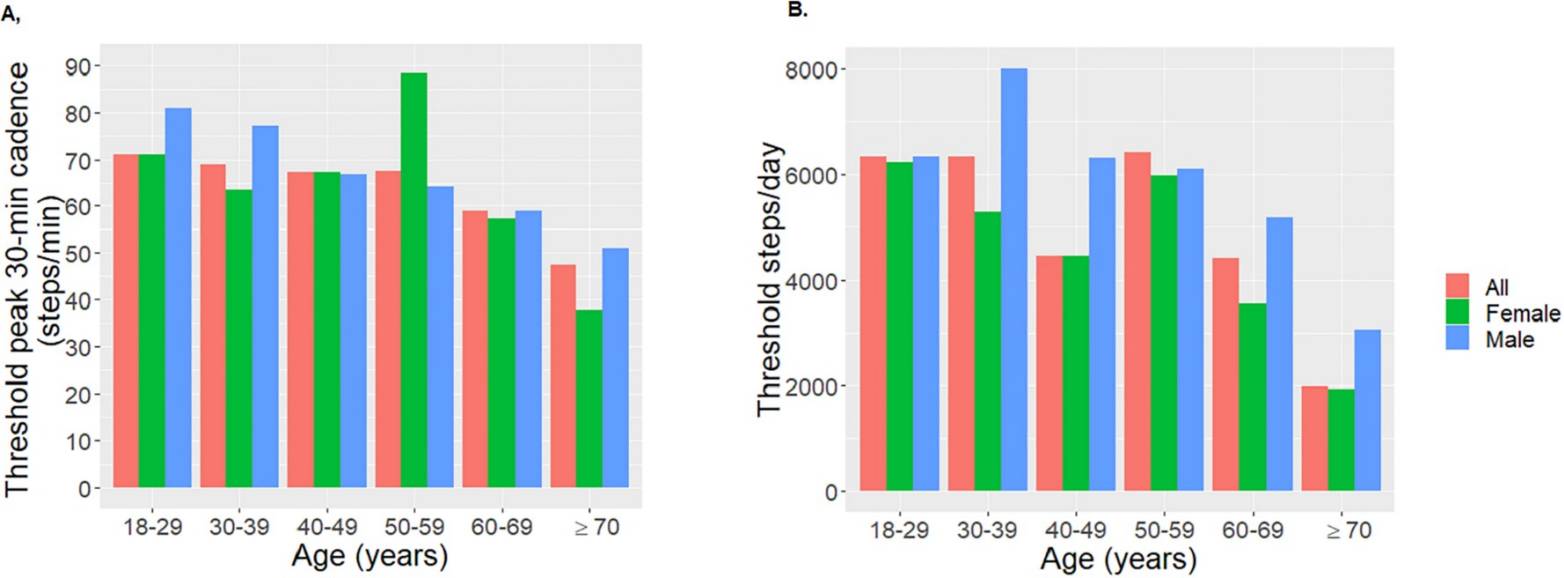
Sex-specific and total population thresholds for metabolic syndrome determined for each age decade. Higher thresholds indicate higher steps/min or more steps/day are required to achieve positive health outcomes. A. Thresholds for peak 30-min cadence B. Thresholds for steps/day.

Fig 1 provides peak 30-min cadence and steps/day thresholds for the sex-specific and combined full datasets, and steps/day thresholds for males and females (Panel A and B respectively). Comprehensive statistical results (e.g., each age-respective AUC and confidence intervals) are provided in S1, S2, S3 and S4 Tables. Higher thresholds indicate that higher intensity (steps/min) and/or more steps/day are required to reach an absence of metabolic health disease. Higher thresholds were observed in men compared to women. In men, higher steps/day thresholds were observed in age ranges of 30–39, while in women, higher thresholds were observed in the age-range 50–59 years. Peak 30-min cadence thresholds were highest for men in the 18–39 year age range, while thresholds for women were highest in the 50–59 year age range. Thresholds decline in older age bins, however, the sample sizes also decline as age increases.

#### Decision tree analysis

**Fig 2:**
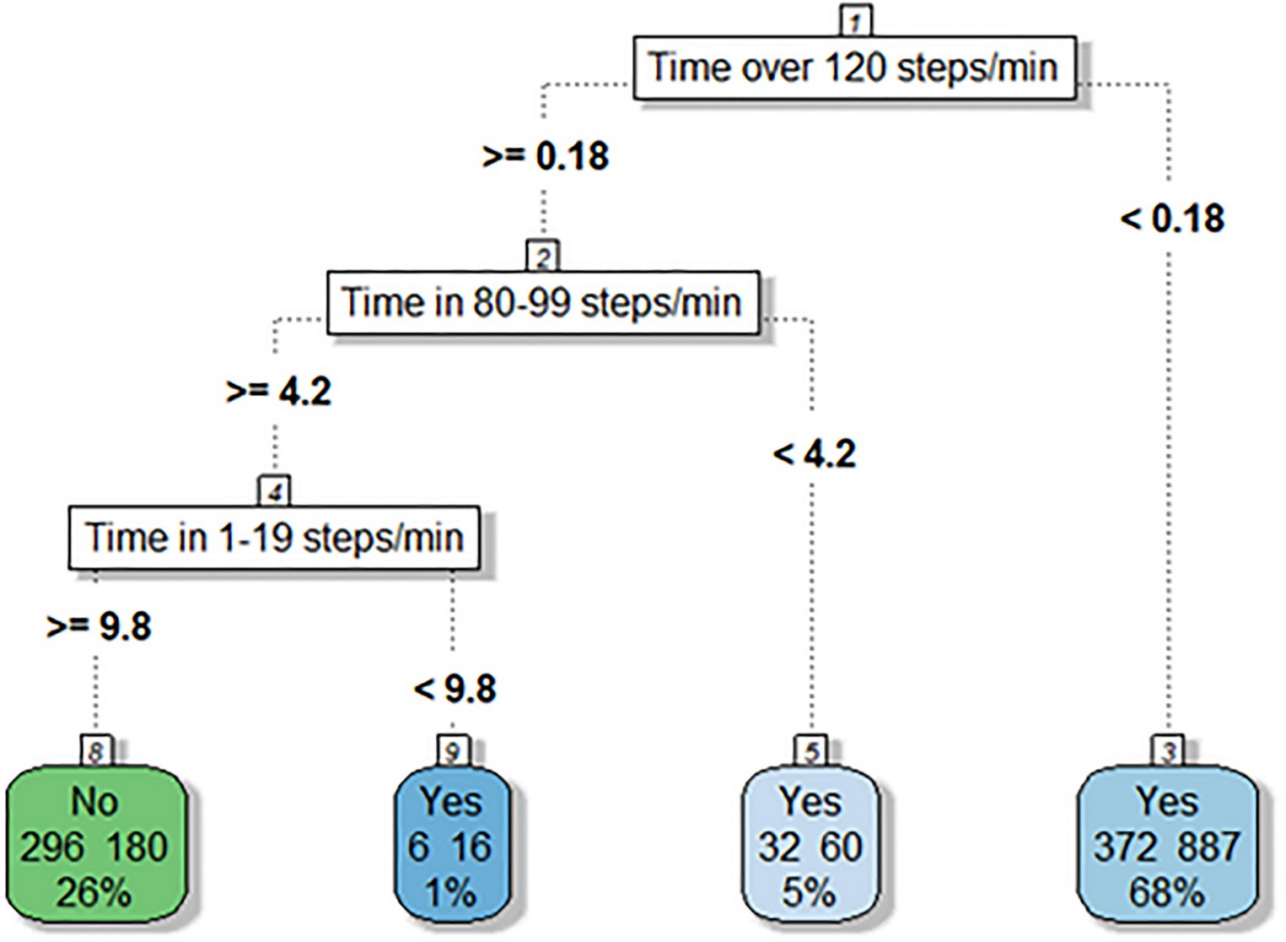
Decision tree classifying low-risk metabolic syndrome by thresholds of time spent in minutes in each cadence band. The value “No” represents absence of risk and “Yes” represents presence of risk. In each box, the values on the left are the number of participants in the box that did not have the risk factor and the value on the right are the number of participants who did have the risk factor. The percentage represents what percent of the total population were contained in the box. Spending virtually no time in cadence band 7 (120 steps/min) would result in a person being classified as at-risk. The only pathway that exists to be classified as not being at-risk is to spend time in cadence band 7 (120 steps/min), ≥4.2 minutes in cadence band 5 (80–99 steps/min), and ≥9.8 minutes in cadence band 1 (1–19 steps/min).

Fig 2 represents the decision tree result for low-risk metabolic syndrome. In Fig 2, spending virtually any time in the seventh cadence band (≥ 120 steps/min), ≥ 4.2 minutes in the fifth cadence band (80–99 steps/min), and ≥ 9.8 minutes in the first cadence band (1–19 steps/min) would result in a person being classified as not at low-risk metabolic syndrome. Additionally, a person who does not spend any time in the seventh cadence band (≥ 120 steps/min) would be classified as at-risk. When the observations were split into 1000 different training sets with 80% of the data reserved for training and 20% for testing, the tree correctly predicted an average of 66.3% of the locations in the test data sets.

The tree had one leaf with this initial data split that categorized the initial split variable into a high probability of absence of a low metabolic syndrome risk factor. The other branches of the decision tree suggest a pathway to absence of risk by spending longer time in lower cadence bands. For example, in Fig 2, spending virtually no time in cadence band 7 (120 steps/min) would result in a person being classified as at-risk. The only risk-free pathway is by spending any time in cadence band 7 (120 steps/min), over 4.2 minutes in cadence band 5 (80–99 steps/min), and over 9.8 minutes in cadence band 1 (1–19 steps/min).

## Discussion

Previous work has been published on associations between cardiometabolic risk factors, steps/day and peak 30-min cadence [11, 21–23]. In the present analysis of the 2005–2006 NHANES accelerometer data, we extend this foundational work by deriving minimum thresholds for peak 30-min cadence and steps/day that classify cardiometabolic risk through a ROC analysis. These thresholds are readily interpretable; steps/day or peak 30-min cadence values below each respective threshold are associated with the presence of cardiometabolic risk. We also derived thresholds for daily time accumulated in defined cadence bands associated with the absence of cardiometabolic risk factors using a decision tree analysis. These thresholds can be interpreted as goals associated with the absence of cardiometabolic risk factors.

### Peak 30-min cadence and steps/day thresholds for cardiometabolic risk

Peak 30-min cadence thresholds across all cardiometabolic risk factors were between 66–72 steps/min. Average national peak 30-min cadence calculated from these same data is 71 steps/min [9], which is within this range. Although these data are cross-sectional and therefore prevent clear conclusions about causality, the results suggest that public health efforts made to elevate peak 30-min cadence beyond these minimal thresholds may be a potent strategy to minimize cardiometabolic risk. Though there are many ways to achieve a peak 30-min cadence above 72 steps/min, the most straightforward method to achieve this target would be to ambulate at a cadence ≥72 steps/min for 30 minutes each day.

Volume thresholds ranged between 4325–6192 steps/day. These thresholds appear lower than the 7,100 to 11,000 steps/day range that has been mapped to assembled studies of objectively-determined 30 minutes of daily moderate-to-vigorous intensity physical activity, which represents the current physical activity standards [24]. There are two reasons for this discrepancy. The first is that our thresholds represent a minimum bar. The threshold is the lowest value that one can achieve before the presence of cardio-metabolic risk. Thus, the threshold should not be considered as a physical activity goal. Recent research that identifies thresholds associated with mortality found similar lower bounds [25]. Second, the benchmark accumulation of 150 minutes per week of moderate-to-vigorous intensity physical activity itself is a product of years of research primarily based on self-reported behavior [26] and a literal translation to objectively monitored time has been questioned [27]. In contrast, the thresholds generated herein are not shaped by any form of preconceived notion related to duration or intensity. Instead they represent minimum objectively-monitored bounds emerging directly from the data set that are associated with a variety of accepted cardiometabolic risk factors.

An AUC over 0.50 indicates the classifier model performs better than a random classifier. The closer the AUC is to 1, the better the classification. While all of the AUCs for both peak 30-min cadence and steps/day were over the value of 0.50, the signals derived from each were not overly strong. The highest AUC was 0.69. The weak signals may be due to several reasons. First, energy expenditure and physical activity are both known to exhibit biological variability and are dependent on many factors (e.g., sex, age, mass, etc.), some of which may not be fully understood (for example, genetic variability) [28]. Second, the step data derived from accelerometer-based devices itself is known to exhibit measurement error when compared to actual steps taken [29]. For example, Toth et al., demonstrated that device accuracy relative to the criterion measure of stepping (direct observation; hand count) varied between ~5 and 120% mean absolute percent error (MAPE) under free-living conditions, depending on the device, wear location, and the step detection algorithm/data processing techniques employed [29]. Thus, studies employing different wearable technologies including different brands of accelerometers and pedometers to determine the relationships between steps/day and cardiometabolic risk may produce varying results. Even with these considerations, the results here demonstrate that while peak 30-min cadence and steps/day only serve as estimated proxies for quality and quantity of physical activity, they are still associated with components of metabolic syndrome. There are also other proxies for habitual physical activity intensity, for example steps accumulated over 10-minute bouts. However, when we plot the highest number of steps over 10-minute bouts against peak 30-min cadence (S2 Fig), there is a strong correlation (r = 0.90). Finally, examining time spent in cadence bands, as we have performed in the decision tree analysis, provides analysis without collapsing the data first.

In addition to our initial evaluations using step-based metrics, future work could derive thresholds for other markers physical activity (e.g., sedentary time, and light, moderate and vigorous PA) and compare them to our findings with peak 30-min cadence[27].

### Age dependent thresholds for cardiometabolic risk

We demonstrate here through a logistic regression model that the estimation of cardiometabolic risk from peak 30-min cadence is age dependent. These findings were further confirmed through the ROC analysis performed in 10-year age ranges. Interestingly, the peak 30-min cadence and steps/day thresholds were highest for women between 40–59 years of age. The interpretation of this finding is that the quality and quantity of daily activity needs to be higher in this age range in order to avoid the presence of cardiometabolic risk. This surprising finding is supported by a study that performed energy balance measurements in perimenopausal women who were within the age range identified in our study [30]. The investigators of that study reported decreased energy expenditure and fat oxidation in women at the onset of menopause, concluding that during this life transition women need to increase their physical activity and/or decrease their energy intake to maintain body weight.

The increased steps/day thresholds in males between the ages of 30–39 were surprising. To our knowledge, there have been no studies examining changes in energy expenditure or energy balance at key time points in males like the ones performed in females [30]. Our findings suggest more research on longitudinal energy expenditure/balance changes in males is warranted.

### Decision tree classification

The decision tree analysis performed herein demonstrated that time accumulated at higher cadence bands resulted in a lower probability of cardiometabolic risk factors. Steps taken in the higher cadence bands are thought to represent increasingly more purposeful movement patterns [8]. Despite strong evidence for the cardiometabolic benefits associated with light intensity physical activity [31], this finding reinforces the additional importance of time spent accumulating these more purposefully higher intensity cadences. On the other hand, there was also a leaf on the decision tree associated with sedentary behavior that led to paths with high-risk, accentuating the relationship between less movement and high cardiometabolic risk. Regardless, the decision tree analysis presented here has several limitations. The first is that the variable (i.e., time spent in cadence band 7 at ≥ 120 steps/min) determined by the algorithm in the R program for the initial split cannot directly be considered the most important factor in determining risk. A deeper analysis that forces splits may be required because the data herein may not allow for sufficient splits at the lower cadences. Despite these concerns, this initial decision tree analysis does provide more rigorous and quantitative step-based goals that effectively delineate healthy populations from those at risk in terms of cardio metabolic health.

This study is the first to calculate volume and effort-specific step-based thresholds for cardiometabolic health-risk stratification. Though there is ample evidence showing a clear dose-response relationship between walking behavior and health [11, 32], no such reports have been presented using step-based physical activity intensity or effort-related metrics. In light of the widely-recognized ease of interpreting physical activity recommendations based upon steps/day and the need for step-based intensity guidelines [6], this novel analysis offers a utilitarian platform from which we may continue to build and advance empirical support for step-based public health recommendations.

## Supporting information

S1 Table Peak 30-min cadence, AUC and thresholds to classify each of the known high-risk metabolic syndrome. Peak 30-min cadence above the threshold classifies positive health outcomes.
(DOCX)

S2 Table Peak 30-min cadence, AUC and thresholds to classify each of the known low-risk metabolic syndrome. Peak 30-min cadence above the threshold classifies positive health outcomes.
(DOCX)

S3 Table Total steps/day, AUC and thresholds to classify each of the known high-risk metabolic syndrome. Total steps/day above the threshold classifies positive health outcomes.
(DOCX)

S4 Table Total steps/day, AUC and thresholds to classify each of the known low-risk metabolic syndrome. Total steps/day above the threshold classifies positive health outcomes.
(DOCX)

S5 Table AIC for logistic regression models. (DOCX)

S1 Fig Plot of steps/min with the PAM’s activity-count based intensity output. When cadence exceeds 180 steps/min, the step count increases as the activity-count based intensity decreases, which is implausible.
(DOCX)

S2 Fig Steps accumulated in 10-minute bouts versus peak 30-min cadence. To calculate bouts, each participant’s 10-minute bout total steps were calculated by summing the top 3 non-overlapping total steps taken in a 10-minute consecutive time interval. Each valid wear day was used in calculating the mean total steps taken in the top 3 x 10-minute bouts.
(DOCX)

## Abbreviations

AUC: area under the curve
BMI: body mass index
PAM: physical activity monitor
ROC: receiver operating characteristic
