# Supplementary material for: Cardiometabolic thresholds for peak 30-min cadence and steps/day
Source: PLoS One. 2019 Aug 2;14(8):e0219933. doi: 10.1371/journal.pone.0219933 (PMC6677301; doi:10.1371/journal.pone.0219933)
Supplement: S1 Table — Peak 30-min cadence above the threshold classifies positive health outcomes. (DOCX) [file pone.0219933.s001.docx]

**Table 1:** Peak 30-min cadence, AUC and thresholds to classify each of the known high-risk metabolic syndrome. Peak 30-min cadence above the threshold classifies positive health outcomes.

| AGE | Gender | AUC | AUC CI | Cut-Point | Cut-Point CI | Specificity | Sensitivity | Controls | Cases |
| --- | --- | --- | --- | --- | --- | --- | --- | --- | --- |
| 18-29 | Male | 0.67 | [0.52, 0.82] | 80.85 | [53.91, 84.64] | 0.50 | 0.80 | 212 | 10 |
| 18-29 | Female | 0.74 | [0.61, 0.87] | 71.20 | [56.12, 71.33] | 0.59 | 0.88 | 227 | 8 |
| 30-39 | Male | 0.50 | [0.32, 0.68] | 77.00 | [76.15, 86.41] | 0.52 | 0.62 | 140 | 13 |
| 30-39 | Female | 0.88 | [0.74, 1.00] | 63.34 | [41.39, 63.46] | 0.75 | 1.00 | 149 | 3 |
| 40-49 | Male | 0.74 | [0.56, 0.92] | 66.69 | [61.37, 72.35] | 0.80 | 0.75 | 166 | 12 |
| 40-49 | Female | 0.77 | [0.65, 0.9] | 67.21 | [60.63, 74.93] | 0.72 | 0.79 | 133 | 14 |
| 50-59 | Male | 0.76 | [0.65, 0.87] | 64.12 | [60.34, 73.99] | 0.78 | 0.69 | 126 | 13 |
| 50-59 | Female | 0.49 | [0.31, 0.67] | 88.27 | [62.54, 92.63] | 0.80 | 0.40 | 100 | 15 |
| 60-69 | Male | 0.53 | [0.38, 0.68] | 58.85 | [56.89, 71.74] | 0.72 | 0.50 | 130 | 20 |
| 60-69 | Female | 0.71 | [0.60, 0.82] | 57.27 | [48.49, 62.09] | 0.64 | 0.78 | 92 | 27 |
| > 70 | Male | 0.55 | [0.42, 0.68] | 51.12 | [38.85, 52.07] | 0.53 | 0.62 | 165 | 21 |
| > 70 | Female | 0.60 | [0.48, 0.72] | 37.95 | [28.77, 52.2] | 0.55 | 0.64 | 102 | 22 |
| 18-29 | All | 0.70 | [0.59, 0.80] | 71.20 | [58.42, 80.9] | 0.65 | 0.61 | 439 | 18 |
| 30-39 | All | 0.55 | [0.39, 0.72] | 68.92 | [56.04, 83.04] | 0.64 | 0.50 | 289 | 16 |
| 40-49 | All | 0.76 | [0.66, 0.86] | 67.21 | [63.45, 72.28] | 0.75 | 0.77 | 299 | 26 |
| 50-59 | All | 0.62 | [0.5, 0.73] | 67.52 | [60.57, 75.22] | 0.65 | 0.61 | 226 | 28 |
| 60-69 | All | 0.65 | [0.56, 0.74] | 58.85 | [56.26, 65.02] | 0.67 | 0.66 | 222 | 47 |
| > 70 | All | 0.59 | [0.5, 0.67] | 47.38 | [37.59, 51.95] | 0.57 | 0.60 | 267 | 43 |
