# Supplementary material for: Cardiometabolic thresholds for peak 30-min cadence and steps/day
Source: PLoS One. 2019 Aug 2;14(8):e0219933. doi: 10.1371/journal.pone.0219933 (PMC6677301; doi:10.1371/journal.pone.0219933)
Supplement: S2 Table — Peak 30-min cadence above the threshold classifies positive health outcomes. (DOCX) [file pone.0219933.s002.docx]

**Table 2:** Peak 30-min cadence, AUC and thresholds to classify each of the known low-risk metabolic syndrome. Peak 30-min cadence above the threshold classifies positive health outcomes.

| AGE | Gender | AUC | AUC CI | Cut-Point | Cut-Point CI | Specificity | Sensitivity | Controls | Cases |
| --- | --- | --- | --- | --- | --- | --- | --- | --- | --- |
| 18-29 | Male | 0.57 | [0.48, 0.66] | 77.53 | [71.61, 89.11] | 0.61 | 0.49 | 102 | 71 |
| 18-29 | Female | 0.62 | [0.53, 0.70] | 71.22 | [67.33, 79.25] | 0.62 | 0.64 | 134 | 55 |
| 30-39 | Male | 0.53 | [0.43, 0.64] | 72.82 | [69.39, 78.42] | 0.46 | 0.68 | 46 | 103 |
| 30-39 | Female | 0.71 | [0.61, 0.80] | 70.35 | [61.85, 71.42] | 0.62 | 0.73 | 87 | 45 |
| 40-49 | Male | 0.68 | [0.59, 0.77] | 75.27 | [69.57, 84.84] | 0.67 | 0.60 | 48 | 120 |
| 40-49 | Female | 0.63 | [0.53, 0.72] | 73.45 | [67.83, 79.15] | 0.66 | 0.60 | 61 | 82 |
| 50-59 | Male | 0.58 | [0.47, 0.69] | 77.47 | [71.65, 82.16] | 0.52 | 0.64 | 40 | 104 |
| 50-59 | Female | 0.63 | [0.53, 0.73] | 68.51 | [62.91, 78.90] | 0.65 | 0.58 | 52 | 74 |
| 60-69 | Male | 0.59 | [0.47, 0.71] | 75.15 | [58.95, 77.65] | 0.48 | 0.71 | 27 | 120 |
| 60-69 | Female | 0.64 | [0.53, 0.75] | 58.01 | [52.25, 69.36] | 0.68 | 0.59 | 37 | 112 |
| > 70 | Male | 0.59 | [0.47, 0.71] | 58.97 | [48.03, 74.17] | 0.52 | 0.68 | 40 | 154 |
| > 70 | Female | 0.61 | [0.49, 0.73] | 40.94 | [34.91, 58.13] | 0.62 | 0.62 | 32 | 103 |
| 18-29 | All | 0.58 | [0.52, 0.64] | 71.22 | [71.04, 82.42] | 0.68 | 0.48 | 236 | 126 |
| 30-39 | All | 0.53 | [0.46, 0.60] | 74.74 | [68.11, 81.18] | 0.50 | 0.55 | 133 | 148 |
| 40-49 | All | 0.65 | [0.58, 0.71] | 73.68 | [69.44, 77.57] | 0.67 | 0.58 | 109 | 202 |
| 50-59 | All | 0.59 | [0.52, 0.67] | 77.75 | [68.57, 79.19] | 0.51 | 0.69 | 92 | 178 |
| 60-69 | All | 0.61 | [0.53, 0.70] | 59.10 | [57.37, 76.77] | 0.69 | 0.48 | 64 | 232 |
| > 70 | All | 0.58 | [0.50, 0.67] | 58.13 | [40.44, 67.06] | 0.47 | 0.70 | 72 | 257 |
