# Supplementary material for: Cardiometabolic thresholds for peak 30-min cadence and steps/day
Source: PLoS One. 2019 Aug 2;14(8):e0219933. doi: 10.1371/journal.pone.0219933 (PMC6677301; doi:10.1371/journal.pone.0219933)
Supplement: S3 Table — Total steps/day above the threshold classifies positive health outcomes. (DOCX) [file pone.0219933.s003.docx]

**Table 3:** Total steps/day, AUC and thresholds to classify each of the known high-risk metabolic syndrome. Total steps/day above the threshold classifies positive health outcomes.

| AGE | Gender | AUC | AUC CI | Cut-Point | Cut-Point CI | Specificity | Sensitivity | Controls | Cases |
| --- | --- | --- | --- | --- | --- | --- | --- | --- | --- |
| 18-29 | Male | 0.66 | [0.46, 0.86] | 6350.08 | [3839.75, 7801.94] | 0.70 | 0.60 | 212 | 10 |
| 18-29 | Female | 0.68 | [0.52, 0.84] | 6240.24 | [2420.07, 6754.62] | 0.47 | 0.88 | 227 | 8 |
| 30-39 | Male | 0.48 | [0.31, 0.66] | 8009.07 | [6642.50, 8722.54] | 0.55 | 0.62 | 140 | 13 |
| 30-39 | Female | 0.62 | [0.23, 1.00] | 5285.36 | [2646.62, 6924.25] | 0.60 | 0.67 | 149 | 3 |
| 40-49 | Male | 0.80 | [0.68, 0.93] | 6322.60 | [4136.80, 8264.29] | 0.78 | 0.67 | 166 | 12 |
| 40-49 | Female | 0.74 | [0.6, 0.88] | 4450.07 | [4119.00, 6511.01] | 0.85 | 0.64 | 133 | 14 |
| 50-59 | Male | 0.77 | [0.65, 0.89] | 6109.54 | [3896.27, 6683.62] | 0.68 | 0.77 | 126 | 13 |
| 50-59 | Female | 0.46 | [0.29, 0.64] | 5986.77 | [5242.88, 8245.68] | 0.53 | 0.53 | 100 | 15 |
| 60-69 | Male | 0.63 | [0.5, 0.77] | 5180.00 | [4361.54, 6330.11] | 0.65 | 0.65 | 130 | 20 |
| 60-69 | Female | 0.70 | [0.59, 0.81] | 3540.44 | [2319.86, 4243.08] | 0.64 | 0.70 | 92 | 27 |
| > 70 | Male | 0.55 | [0.43, 0.67] | 3050.64 | [1607.93, 4338.43] | 0.47 | 0.67 | 165 | 21 |
| > 70 | Female | 0.63 | [0.51, 0.75] | 1923.32 | [1212.58, 2081.09] | 0.57 | 0.73 | 102 | 22 |
| 18-29 | All | 0.66 | [0.52, 0.79] | 6347.18 | [3795.14, 6756.98] | 0.57 | 0.72 | 439 | 18 |
| 30-39 | All | 0.56 | [0.4, 0.71] | 6341.66 | [6332.16, 12225.20] | 0.49 | 0.75 | 289 | 16 |
| 40-49 | All | 0.78 | [0.69, 0.87] | 4450.07 | [4123.20, 7608.24] | 0.89 | 0.62 | 299 | 26 |
| 50-59 | All | 0.66 | [0.55, 0.77] | 6414.50 | [4021.29, 6726.86] | 0.56 | 0.71 | 226 | 28 |
| 60-69 | All | 0.69 | [0.6, 0.77] | 4412.95 | [3540.44, 5382.07] | 0.64 | 0.70 | 222 | 47 |
| > 70 | All | 0.60 | [0.52, 0.69] | 1975.57 | [1411.45, 3049.36] | 0.62 | 0.58 | 267 | 43 |
