# Supplementary material for: Cardiometabolic thresholds for peak 30-min cadence and steps/day
Source: PLoS One. 2019 Aug 2;14(8):e0219933. doi: 10.1371/journal.pone.0219933 (PMC6677301; doi:10.1371/journal.pone.0219933)
Supplement: S4 Table — Total steps/day above the threshold classifies positive health outcomes. (DOCX) [file pone.0219933.s004.docx]

**Table 4:** Total steps/day, AUC and thresholds to classify each of the known low-risk metabolic syndrome. Total steps/day above the threshold classifies positive health outcomes.

| AGE | Gender | AUC | AUC CI | Cut-Point | Cut-Point CI | Specificity | Sensitivity | Controls | Cases |
| --- | --- | --- | --- | --- | --- | --- | --- | --- | --- |
| 18-29 | Male | 0.52 | [0.43, 0.61] | 7318.32 | [6148.21, 9687.64] | 0.51 | 0.56 | 102 | 71 |
| 18-29 | Female | 0.48 | [0.39, 0.57] | 5450.45 | [5420.00, 6398.52] | 0.45 | 0.62 | 134 | 55 |
| 30-39 | Male | 0.53 | [0.42, 0.63] | 8627.71 | [6147.89, 8660.83] | 0.63 | 0.49 | 46 | 103 |
| 30-39 | Female | 0.64 | [0.53, 0.74] | 5460.30 | [4904.27, 5910.40] | 0.60 | 0.67 | 87 | 45 |
| 40-49 | Male | 0.68 | [0.59, 0.77] | 8335.21 | [7424.71, 8964.99] | 0.62 | 0.69 | 48 | 120 |
| 40-49 | Female | 0.61 | [0.52, 0.71] | 6944.89 | [5499.32, 7076.53] | 0.52 | 0.72 | 61 | 82 |
| 50-59 | Male | 0.56 | [0.45, 0.67] | 6828.45 | [6444.00, 8777.86] | 0.60 | 0.54 | 40 | 104 |
| 50-59 | Female | 0.63 | [0.53, 0.73] | 5422.96 | [4919.38, 6479.55] | 0.67 | 0.58 | 52 | 74 |
| 60-69 | Male | 0.61 | [0.49, 0.72] | 4741.52 | [4404.61, 10046.32] | 0.74 | 0.41 | 27 | 120 |
| 60-69 | Female | 0.64 | [0.53, 0.75] | 4000.50 | [3977.20, 5819.33] | 0.68 | 0.64 | 37 | 112 |
| > 70 | Male | 0.61 | [0.49, 0.73] | 4546.49 | [3930.43, 5671.90] | 0.52 | 0.77 | 40 | 154 |
| > 70 | Female | 0.58 | [0.47, 0.70] | 1952.29 | [1409.55, 3039.09] | 0.62 | 0.57 | 32 | 103 |
| 18-29 | All | 0.52 | [0.46, 0.58] | 6200.85 | [5410.33, 7824.57] | 0.47 | 0.61 | 236 | 126 |
| 30-39 | All | 0.54 | [0.48, 0.61] | 6697.80 | [6332.16, 6968.89] | 0.60 | 0.55 | 133 | 148 |
| 40-49 | All | 0.62 | [0.55, 0.68] | 7425.02 | [6683.88, 8293.74] | 0.61 | 0.62 | 109 | 202 |
| 50-59 | All | 0.57 | [0.50, 0.64] | 6439.21 | [5351.51, 7436.50] | 0.55 | 0.56 | 92 | 178 |
| 60-69 | All | 0.61 | [0.53, 0.68] | 4604.70 | [3871.29, 5807.64] | 0.66 | 0.54 | 64 | 232 |
| > 70 | All | 0.58 | [0.50, 0.67] | 3153.82 | [1952.29, 5344.79] | 0.49 | 0.64 | 72 | 257 |

**Table 5:** AIC for logistic regression models

| Measurement | AIC | Measurement | AIC |
| --- | --- | --- | --- |
| Low-risk Waist Circumference | | | |
| Peak 30-minute cadence | 3650.02 | Steps/day | 3649.54 |
| Peak 30-minute cadence adjusted for smoking | 1720.24 | Steps/day adjusted for smoking | 1709.20 |
| Peak 30-minute cadence adjusted for age | 3552.47 | Steps/day adjusted for age | 3552.09 |
| Peak 30-minute cadence adjusted for age and smoking | 1692.64 | Steps/day adjusted for age and smoking | 1684.45 |
| High-risk Waist Circumference | | | |
| Peak 30-minute cadence | 4088.74 | Steps/day | 4008.10 |
| Peak 30-minute cadence adjusted for smoking | 1802.37 | Steps/day adjusted for smoking | 1754.88 |
| Peak 30-minute cadence adjusted for age | 4080.28 | Steps/day adjusted for age | 4003.85 |
| Peak 30-minute cadence adjusted for age and smoking | 1804.10 | Steps/day adjusted for age and smoking | 1754.26 |
| Low-risk Blood Pressure | | | |
| Peak 30-minute cadence | 3269.15 | Steps/day | 3318.01 |
| Peak 30-minute cadence adjusted for smoking | 1545.03 | Steps/day adjusted for smoking | 1563.89 |
| Peak 30-minute cadence adjusted for age | 3083.02 | Steps/day adjusted for age | 3090.73 |
| Peak 30-minute cadence adjusted for age and smoking | 1489.15 | Steps/day adjusted for age and smoking | 1495.78 |
| High-risk Blood Pressure | | | |
| Peak 30-minute cadence | 2828.97 | Steps/day | 2893.75 |
| Peak 30-minute cadence adjusted for smoking | 1372.30 | Steps/day adjusted for smoking | 1412.025 |
| Peak 30-minute cadence adjusted for age | 2626.64 | Steps/day adjusted for age | 2648.77 |
| Peak 30-minute cadence adjusted for age and smoking | 1304.27 | Steps/day adjusted for age and smoking | 1325.82 |
| Low-risk Metabolic Syndrome | | | |
| Peak 30-minute cadence | 2387.20 | Steps/day | 2445.75 |
| Peak 30-minute cadence adjusted for smoking | 1120.07 | Steps/day adjusted for smoking | 1144.21 |
| Peak 30-minute cadence adjusted for age | 2330.87 | Steps/day adjusted for age | 2371.20 |
| Peak 30-minute cadence adjusted for age and smoking | 1116.36 | Steps/day adjusted for age and smoking | 1137.42 |
| High-risk Metabolic Syndrome | | | |
| Peak 30-minute cadence | 1124.45 | Steps/day | 1112.60 |
| Peak 30-minute cadence adjusted for smoking | 571.02 | Steps/day adjusted for smoking | 564.57 |
| Peak 30-minute cadence adjusted for age | 1119.95 | Steps/day adjusted for age | 1108.91 |
| Peak 30-minute cadence adjusted for age and smoking | 570.68 | Steps/day adjusted for age and smoking | 565.04 |
