# Supplementary material for: Cardiometabolic thresholds for peak 30-min cadence and steps/day
Source: PLoS One. 2019 Aug 2;14(8):e0219933. doi: 10.1371/journal.pone.0219933 (PMC6677301; doi:10.1371/journal.pone.0219933)
Supplement: S5 Table — (DOCX) [file pone.0219933.s005.docx]

**Supplemental Table 5.** AIC for logistic regression models

| Measurement | AIC | Measurement | AIC |
| --- | --- | --- | --- |
| Low-risk Waist Circumference | | | |
| Peak 30-minute cadence | 3650.02 | Steps/day | 3649.54 |
| Peak 30-minute cadence adjusted for smoking | 1720.24 | Steps/day adjusted for smoking | 1709.20 |
| Peak 30-minute cadence adjusted for age | 3552.47 | Steps/day adjusted for age | 3552.09 |
| Peak 30-minute cadence adjusted for age and smoking | 1692.64 | Steps/day adjusted for age and smoking | 1684.45 |
| High-risk Waist Circumference | | | |
| Peak 30-minute cadence | 4088.74 | Steps/day | 4008.10 |
| Peak 30-minute cadence adjusted for smoking | 1802.37 | Steps/day adjusted for smoking | 1754.88 |
| Peak 30-minute cadence adjusted for age | 4080.28 | Steps/day adjusted for age | 4003.85 |
| Peak 30-minute cadence adjusted for age and smoking | 1804.10 | Steps/day adjusted for age and smoking | 1754.26 |
| Low-risk Blood Pressure | | | |
| Peak 30-minute cadence | 3269.15 | Steps/day | 3318.01 |
| Peak 30-minute cadence adjusted for smoking | 1545.03 | Steps/day adjusted for smoking | 1563.89 |
| Peak 30-minute cadence adjusted for age | 3083.02 | Steps/day adjusted for age | 3090.73 |
| Peak 30-minute cadence adjusted for age and smoking | 1489.15 | Steps/day adjusted for age and smoking | 1495.78 |
| High-risk Blood Pressure | | | |
| Peak 30-minute cadence | 2828.97 | Steps/day | 2893.75 |
| Peak 30-minute cadence adjusted for smoking | 1372.30 | Steps/day adjusted for smoking | 1412.025 |
| Peak 30-minute cadence adjusted for age | 2626.64 | Steps/day adjusted for age | 2648.77 |
| Peak 30-minute cadence adjusted for age and smoking | 1304.27 | Steps/day adjusted for age and smoking | 1325.82 |
| Low-risk Metabolic Syndrome | | | |
| Peak 30-minute cadence | 2387.20 | Steps/day | 2445.75 |
| Peak 30-minute cadence adjusted for smoking | 1120.07 | Steps/day adjusted for smoking | 1144.21 |
| Peak 30-minute cadence adjusted for age | 2330.87 | Steps/day adjusted for age | 2371.20 |
| Peak 30-minute cadence adjusted for age and smoking | 1116.36 | Steps/day adjusted for age and smoking | 1137.42 |
| High-risk Metabolic Syndrome | | | |
| Peak 30-minute cadence | 1124.45 | Steps/day | 1112.60 |
| Peak 30-minute cadence adjusted for smoking | 571.02 | Steps/day adjusted for smoking | 564.57 |
| Peak 30-minute cadence adjusted for age | 1119.95 | Steps/day adjusted for age | 1108.91 |
| Peak 30-minute cadence adjusted for age and smoking | 570.68 | Steps/day adjusted for age and smoking | 565.04 |
