# Supplementary material for: Cardiometabolic thresholds for peak 30-min cadence and steps/day
Source: PLoS One. 2019 Aug 2;14(8):e0219933. doi: 10.1371/journal.pone.0219933 (PMC6677301; doi:10.1371/journal.pone.0219933)

**Supplemental Figure 1:** Plot of steps/min with the PAM’s activity-count based intensity output. When cadence exceeds 180 steps/min, the step count increases as the activity-count based intensity decreases, which is implausible.


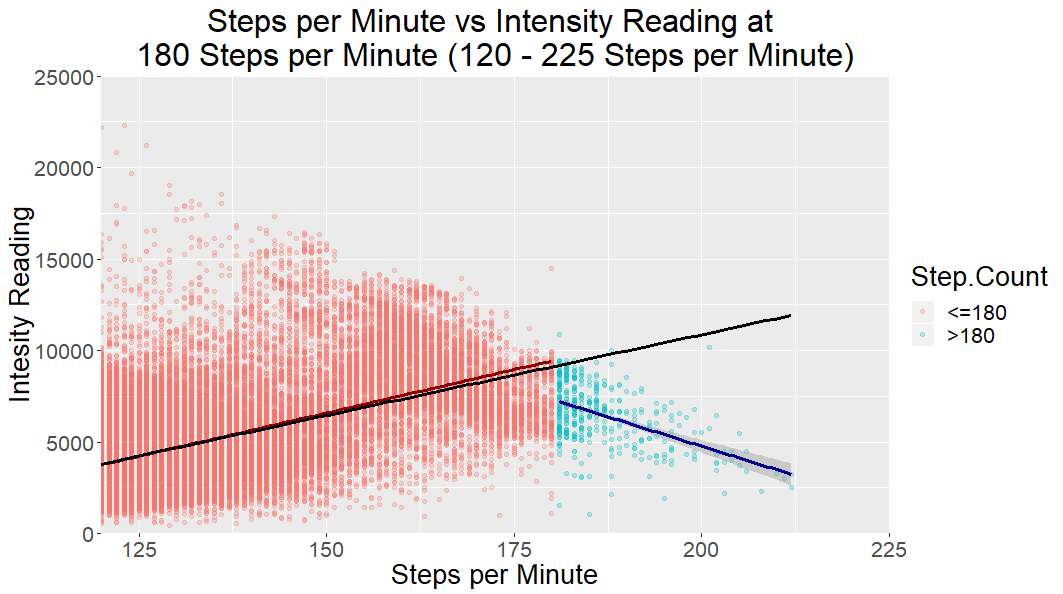

Supplement: S1 Fig — When cadence exceeds 180 steps/min, the step count increases as the activity-count based intensity decreases, which is implausible. (DOCX) [file pone.0219933.s006.docx]
