# Supplementary material for: Cardiometabolic thresholds for peak 30-min cadence and steps/day
Source: PLoS One. 2019 Aug 2;14(8):e0219933. doi: 10.1371/journal.pone.0219933 (PMC6677301; doi:10.1371/journal.pone.0219933)

**Supplemental Figure 2:** Steps accumulated in 10-minute bouts versus peak 30-min cadence. To calculate bouts, each participant’s 10-minute bout total steps were calculated by summing the top 3 non-overlapping total steps taken in a 10-minute consecutive time interval.  Each valid wear day was used in calculating the mean total steps taken in the top 3 x 10-minute bouts.


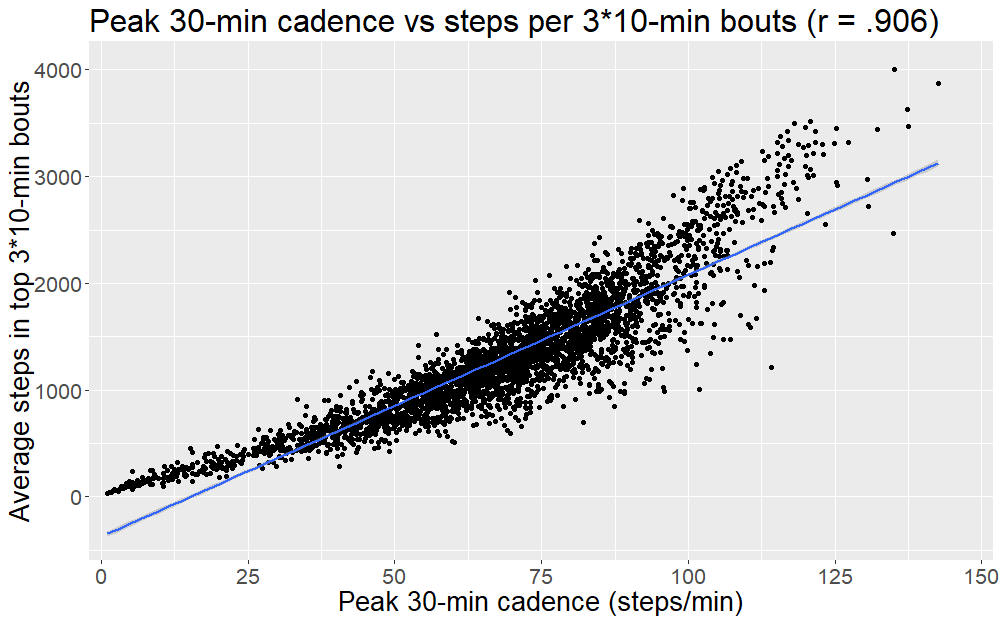

Supplement: S2 Fig — To calculate bouts, each participant’s 10-minute bout total steps were calculated by summing the top 3 non-overlapping total steps taken in a 10-minute consecutive time interval. Each valid wear day was used in calculating the mean total steps taken in the top 3 x 10-minute bouts. (DOCX) [file pone.0219933.s007.docx]
